# “Giant” Nitrogen Uptake in Ionic Liquids Confined in Carbon Pores

**DOI:** 10.1021/jacs.1c00783

**Published:** 2021-06-15

**Authors:** Ipek Harmanli, Nadezda V. Tarakina, Markus Antonietti, Martin Oschatz

**Affiliations:** † Department of Colloid Chemistry, Research Campus Golm, Max Planck Institute of Colloids and Interfaces, Am Mühlenberg 1, 14476 Potsdam, Germany; ‡ Institute of Chemistry, University of Potsdam, Karl-Liebknecht-Strase 24-25, D-14476 Potsdam, Germany

## Abstract

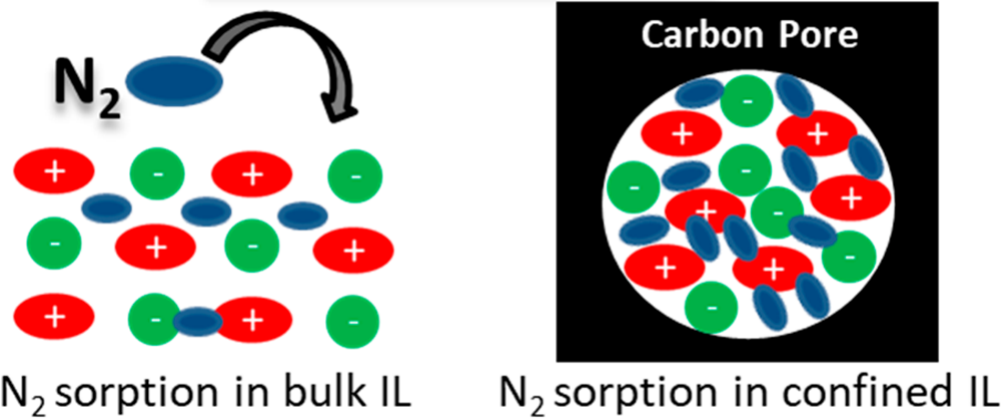

Ionic liquids are well known for their high gas absorption capacity. It is shown that this is not a solvent constant, but can be enhanced by another factor of 10 by pore confinement, here of the ionic liquid (IL) 1-ethyl-3-methylimidazolium acetate (EmimOAc) in the pores of carbon materials. A matrix of four different carbon compounds with micro- and mesopores as well as with and without nitrogen doping is utilized to investigate the influence of the carbons structure on the nitrogen uptake in the pore-confined EmimOAc. In general, the absorption is most improved for IL in micropores and in nitrogen-doped carbon. This effect is so large that it is already seen in TGA and DSC experiments. Due to the low vapor pressure of the IL, standard volumetric sorption experiments can be used to quantify details of this effect. It is reasoned that it is the change of the molecular arrangement of the ions in the restricted space of the pores that creates additional free volume to host molecular nitrogen.

## Introduction

One of the most important physical properties of liquids is their ability to dissolve gases.^1−3^ Obvious examples of the importance of this process are the temperature-dependent solubility of carbon dioxide and oxygen in water, which are crucial for the pH of oceans and life in the hydrosphere of the planet. It is known that the solubility of gases is strongly pressure- and temperature-dependent.^3^ From another point of view, solubility of gases can significantly change if the structure of the solvent changes. For example, weakly interacting gases such as methane or krypton are not soluble in liquid water but can be confined in larger amounts in ice due to the formation of clathrates.^4,5^

In the field of catalysis, the efficiency of oxidation or reduction reactions which are carried out in liquid solvents is in large part determined by the solubility of gases in the reaction media and are thus more efficiently performed at high pressure above the solution, following Henry’s law.^6,7^ One of the most prominent and actual examples is the electrochemical “dream” reduction of nitrogen to ammonia (often referred to as nitrogen reduction reaction, NRR) by using protons and electrons instead of molecular hydrogen as in the industrially applied Haber–Bosch process.^8−11^ More and more catalysts and mechanisms are reported for the activation of the chemically very stable dinitrogen molecule, and ever higher faradaic efficiencies are achieved for this reaction, as the hydrogen evolution reaction can be efficiently suppressed by introducing specific nitrogen-binding and activation sites into the chemical construction of the catalysts.^10,12^ Despite this progress, in all cases the space–time yield of this reaction is meanwhile restricted by the concentration of nitrogen in solution. In the overwhelming majority of reports, aqueous electrolytes are employed, where the solubility of nitrogen is very low (less than 20 mg/L at 1 bar and room temperature). This simply means that the achievable ammonia production rates are limited due to the low concentration of substrate molecules near the catalytically active sites. According to Henry’s law, one way to increase the concentration of dissolved gases in a liquid is to increase its partial pressure in the surrounding gas phase, and application of, for example, 100 bar nitrogen pressure increases the solubility to ∼0.0485 mol/kg or more than 1.3 g/kg (313.15 K).^13^ This, however, compromises the biggest practical advantage of electrochemical NRR, namely, ambient and simple reaction conditions.

Another way is to choose conducting liquids with higher uptake of gas molecules. Ionic liquids (ILs) are an attractive alternative for such solution-based reactions.^14−16^ ILs are molten salts that are beneficial for many applications due to their special ability to be tuned in terms of ionic construction and thus properties.^17−19^ In particular, the gas absorption capacity of different ILs can show significant variations depending on the cations and anions. In simple words, ILs possess a higher polarizability,^20−24^ but also a higher so-called free volume (non-matter-filled sites between the ions) due to the bulky ions and their sterically demanding “frustrated packing”. This, in combination with their ionic conductivity and usually high electrochemical stability window, makes ILs attractive candidates as reaction media for electrocatalytic processes.^25,26^ For instance, McFarlane and co-workers reported NRR under ambient conditions with a faradaic efficiency of 60% by using hydrophobic ILs with high nitrogen uptake in combination with an iron-based catalyst.^27^ Such high faradaic efficiencies can only be achieved in ILs^28^ at low concentrations of water.

The catalyst is usually supported on or even itself a nanoporous material with high surface area which is in contact with the bulk electrolyte to improve the electrochemically active surface area of the heterogeneous system. These pores will be then filled with ionic liquid, and because pores and ionic liquids are comparable, but not commensurable in size, it must be expected that the physicochemical properties of the ILs will change in such confinement (especially in the possible presence of an electric potential).^29^ For example, it is known that the freezing point of water decreases significantly when the molecules are confined into pores of several nanometers in size.^30,31^ In a similar sense, a change of the molecular arrangement of IL ions due to confinement into nanoporous materials may have a significant influence on the solubility of compounds, here in the case of dinitrogen.

The nitrogen absorption in pore-confined ILs and the influences of pore sizes as well as polarity of the pore walls are analyzed within a series of four carbon materials with different pore sizes and different polarity and work function controlled by nitrogen doping. The carbon materials have been loaded with different ratios of the ionic liquid 1-ethyl-3-methylimidazolium acetate (EmimOAc). While the nitrogen solubility is already much higher in the bulk IL as compared to liquid water, it will be shown that the uptake of N_2_ into pore-confined EmimOAc is increased by another order of magnitude and remains reversible at the same time, as shown by nitrogen absorption–desorption measurements. Due to the nonvolatility of ionic liquids, this can be easily followed in a standard volumetric physisorption apparatus.

## Results and Discussion

**Figure 1:**
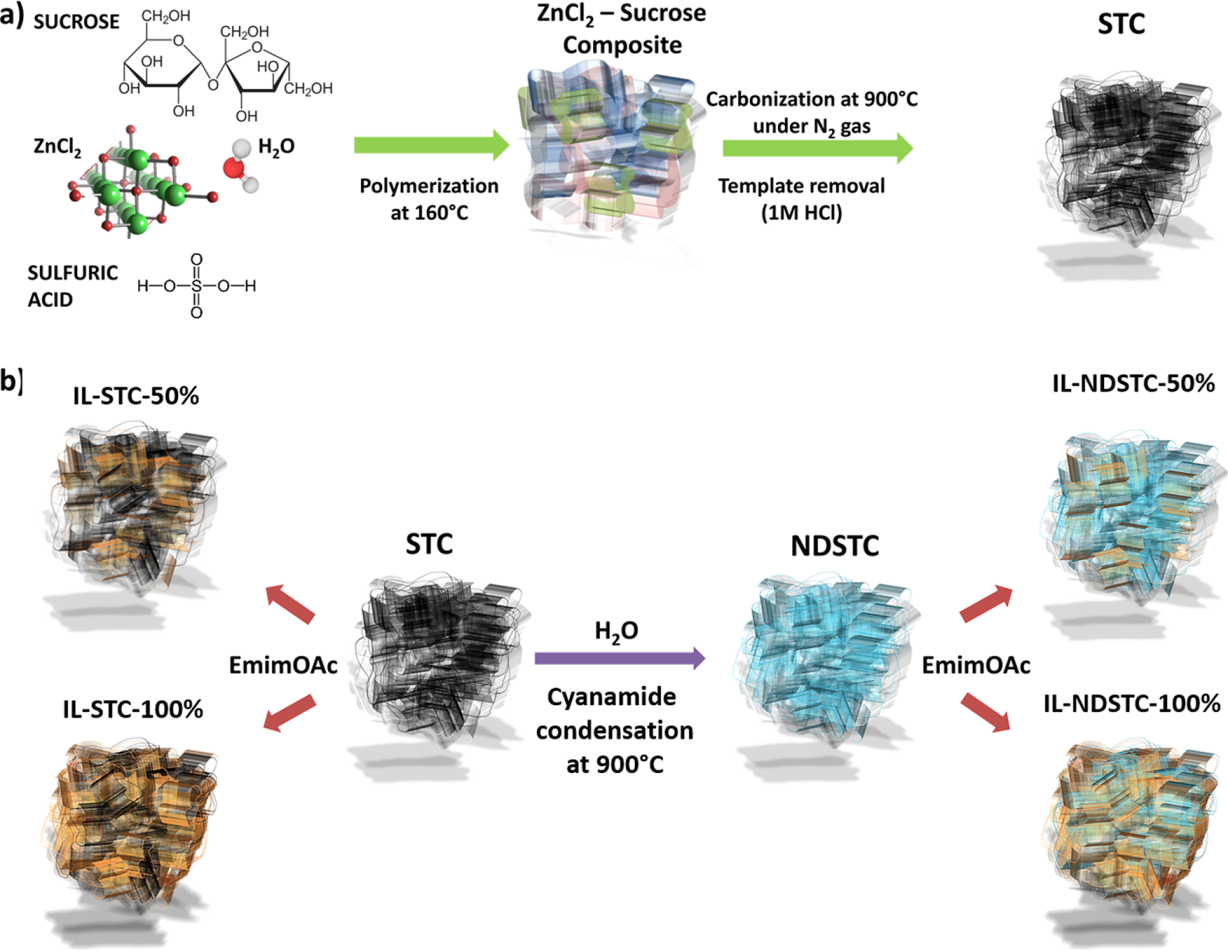
(a) Carbon synthesis by salt-templating and (b) nitrogen doping as well as loading of different amounts of EmimOAc ionic liquid.

A set consisting of four different carbon materials was prepared for the investigation of nitrogen solubility in pore-confined ionic liquids (Figure 1). The parental carbons STC-1 and STC-8, which have different pore structures, were synthesized via the salt-templating method by using sucrose as the carbon precursor and different amounts of ZnCl_2_ as porogen to introduce micropores and mesopores, as described by Yan et al.^32^ The “X” in STC-X refers to the different mass ratios of ZnCl_2_/sucrose used for carbon synthesis. STC-1 and STC-8 were further functionalized with nitrogen heteroatoms by postfunctionalization with cyanamide at 800 °C. This treatment introduces nitrogen heteroatoms covalently bonded to the carbon network in different motifs.^32^ Generally, the presence of electronegative nitrogen atoms induces higher polarity into the carbon pore walls and thus stronger interaction with adsorbates in these pores.^33^ The resulting nitrogen-doped carbons are denoted as NDSTC-1 and NDSTC-8, following their parental materials.

**Figure 2:**
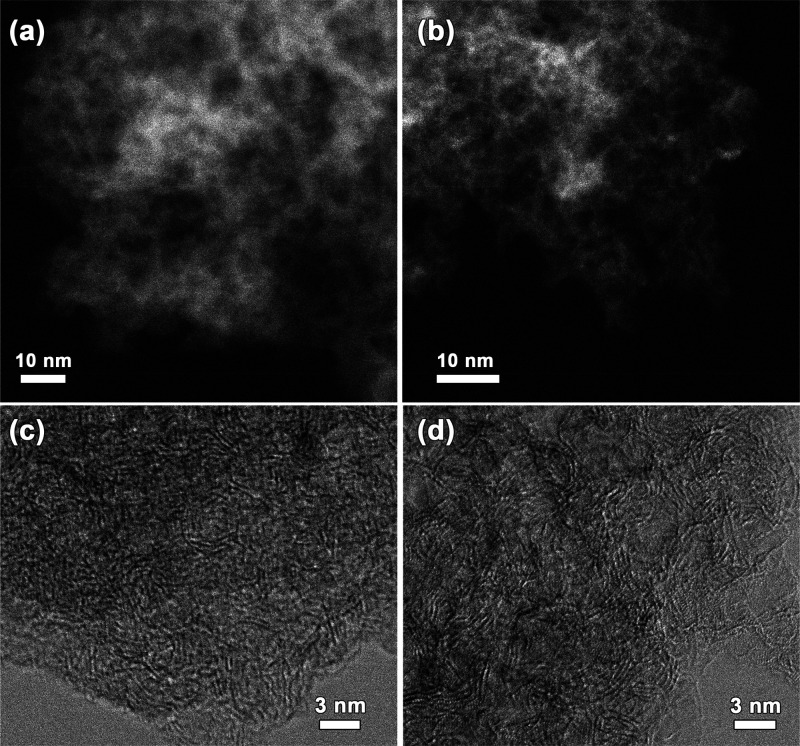
TEM high-resolution (a, b) annular dark-field (ADF) and (c, d) bright-field (BF) STEM images of (a, c) IL-NDSTC-8-100% and (b, d) NDSTC-8 without IL.

Transmission electron microscopy (TEM) and scanning transmission electron microscopy (STEM) images (Figure 2 and Figure S1a–d) show the uniform pore structure of the carbon materials. As expected, larger pores are present in STC-8 and NDSTC-8 as compared to the carbons prepared at a lower salt content. The comparable structures of STC-1 and NDSTC-1 as well as of STC-8 and NDSTC-8 indicate that the nitrogen doping accomplished by postreaction with cyanamide has no significant effect on the carbon microstructure. Scanning electron microscopy (SEM) imaging shows that the single particles additionally contain irregular textural pores with sizes in the micrometer range (exemplarily shown for NDSTC-8 in Figure S2a).

Homogenous distribution of carbon and nitrogen in the materials is confirmed by scanning electron microscopy coupled to energy dispersive X-ray spectroscopy analysis (SEM-EDX) mapping (exemplarily shown for NDSTC-8 in Figure S2b). Elemental analysis (EA) confirms that the nitrogen content was enhanced by cyanamide condensation from values below 1 wt % for STC-1 and STC-8 to 4.0 and 4.5 wt % for NDSTC-1 and NDSTC-8, respectively (Table 1). The presence of those heteroatoms will influence not only polarity but also the interaction with IL ions. In particular, it can be expected that the presence of nitrogen atoms will affect the strength of the interaction between the carbon pore walls and ionic liquid ions.

**Table 1:** Nitrogen Content of the STCs and NDSTCs Determined by Elemental Analysis (*N*_EA_) and SEM-EDX Analysis (*N*_EDX_), BET Specific Surface Area (SSA_BET_), Total Pore Volume (*V*_total_), DFT Micropore Volume (*V*_mic_), and DFT Mesopore Volume (*V*_meso_) Determined from N_2_ Physisorption at 77 K

			N_2_ physisorption (77 K)			
				DFT		
sample	*N*_EA_ (wt %)	*N*_EDX_ (wt %)	*V*_total_ (cm^3^/g)	*V*_mic_ (cm^3^/g)	*V*_meso_ (cm^3^/g)	SSA_BET_ (m^2^/g)
STC-1	0.20		0.82	0.55	0.15	1714
STC-8	0.84		1.40	0.34	0.96	2118
NDSTC-1	4.01		0.62	0.43	0.07	1123
NDSTC-8	4.54	5.67	1.32	0.37	0.84	2086

**Figure 3:**
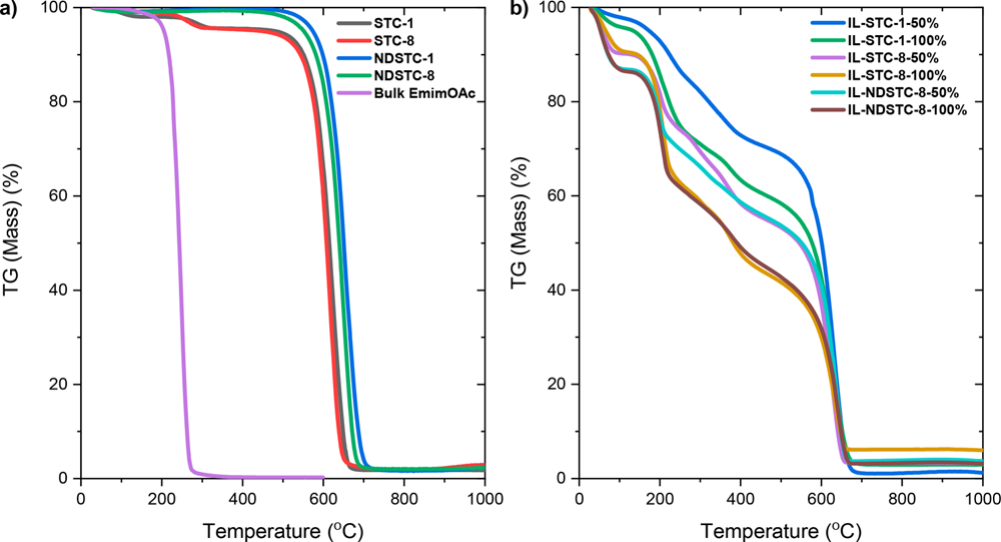
TGA curves under air of (a) STCs, NDSTCs, and bulk EmimOAc ionic liquid and (b) IL-loaded carbon samples with different contents of EmimOAc.

Thermogravimetric analysis (TGA) was applied under air up to 1000 °C in order to analyze the materials for possible inorganic residuals (Figure 3a). NDSTCs and STCs show a sharp mass loss with an onset temperature slightly below 500 °C with <3 wt % ash content after ∼700 °C as expected for synthetic porous carbon with only a minor content of inorganic residues.

**Figure 4:**
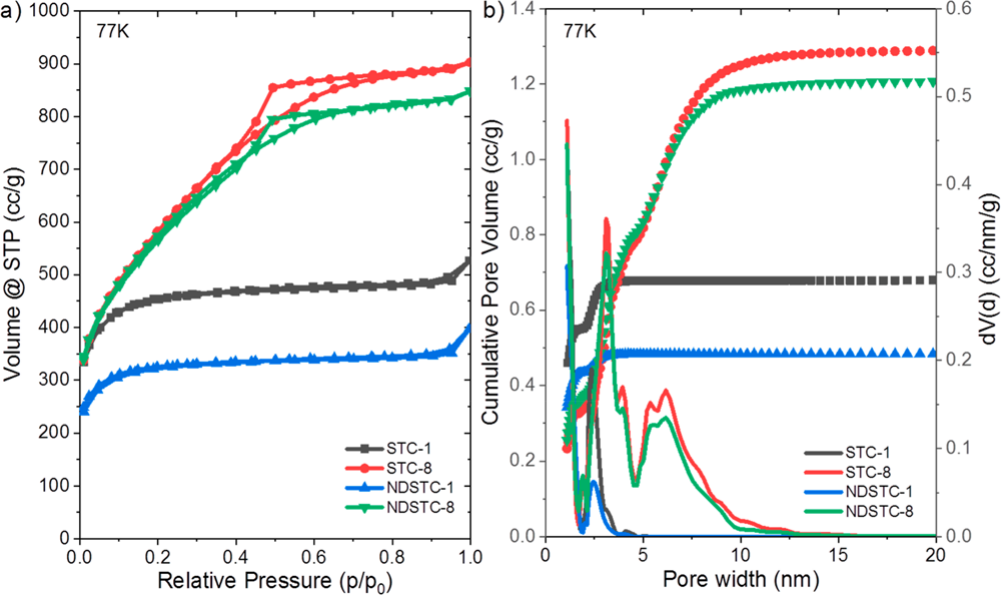
(a) N_2_ physisorption isotherms measured at 77 K and (b) corresponding cumulative (line with symbols) and differential (lines without symbols) QSDFT pore size distributions of the four pristine carbon materials.

N_2_ physisorption measurements of bare STC-1 and STC-8 at 77 K show a typical type I isotherm for STC-1 according to the IUPAC classification and a type IV isotherm for STC-8, which are characteristic for microporous and mesoporous materials, respectively (Figure 4a). In accordance with previous works on salt-templated carbon materials,^19,32^ an increase of the salt content leads to an increase of the specific surface area and the total pore volume (Table 1). The hysteresis loops of STC-8 and NDSTC-8 in the relative pressure range *p*/*p*_0_ = 0.4–0.8 reflect the presence of mesopores in the size range from 3 to 10 nm, and all samples have a high content of micropores below 2 nm in size, as indicated by high volume adsorbed at low pressure and the pore size distributions calculated from quenched solid density functional theory (QSDFT) (Figure 4b). Despite some minor changes in the specific surface areas and total pore volumes, the pore size distributions (PSDs) show that the pore structures generally remain only slightly affected by the nitrogen-doping procedure; that is, it is indeed essentially a wall functionalization process.

On the basis of these experiments, one can understand STC-1, NDSTC-1, STC-8, and NDSTC-8 as a systematic matrix of carbon materials in which the heteroatom content and the pore structure are independently modified. As the samples are all prepared by the same templating approach, at the same temperature, and from similar precursors, differences in the carbon nanostructure should remain limited. According to recent studies, the four model systems, however, indeed show significant differences in their interaction with ionic liquids.^32^

In order to investigate the effect of confinement of IL into carbon pores on the uptake of N_2_, EmimOAc has been infiltrated into the STCs and NDSTCs with two different loadings per sample (50% and 100% bulk IL volume relative to the total pore volume determined from N_2_ physisorption at 77 K). The comparison of TEM images of these IL-loaded carbon samples (Figure 2 and Figure S1e–h) (which can only be done due to the nonvolatility of the ILs) and the pristine carbons shows the homogeneity of IL loading, with only some contrast changes due to smaller density differences. Especially, the mesoporous NDSTC-8 has a less porous appearance after IL loading. In the BF-STEM images of NDSTC-8 the atomic structure is resolved much more clearly than on the same sample loaded with IL (Figure 1), indicating that the IL is incorporated into the pores of the carbon, increasing the mass–thickness contrast and slightly reducing the resolving power on the images. The successful uptake of the IL by the material was also proven by elemental analysis, and expectedly the nitrogen contents increase significantly after addition of the IL for all materials due to the presence of 1-ethyl-3-methylimidazolium cations, as also confirmed by SEM-EDX (Table S1). Nitrogen content also increases with IL loading. SEM-EDX elemental mapping was exemplarily carried out for STC-8 and NDSTC-8 samples with different IL loadings and shows a homogeneous distribution of nitrogen in all samples (Figure S3). Electron energy loss spectroscopy (EELS) elemental mapping of high-resolution ADF STEM images was performed for IL-NDSTC-8-100%. As there is no specific element in the IL that would allow distinguishing the confined liquid from the carbon matrix during elemental analysis, we tried to compare the distribution and quantity of oxygen and nitrogen in the sample (Figure S4). The overall oxygen content of the sample measured with EELS is about 3.5 at. %, and the nitrogen content is about 7.5 at. %. Both elements are distributed nonuniformly in the carbon matrix; however it can be seen that nitrogen is present inside pores and also surrounds the pores, while the oxygen signal is coming only from the inside of the pores, where the IL is located.

Nitrogen physisorption isotherms of the IL-loaded materials at 77 K (Figure S5) show that STC-8-50% and NDSTC-8-50% still have open porosity but with a significantly decreased total pore volume (0.37 cm^3^/g for STC-8-50% and 0.29 cm^3^/g NDSTC-8-50%) and specific surface area (423 m^2^/g for STC-8-50% and 345 m^2^/g NDSTC-8-50%) in comparison to the pristine samples. In the microporous STC-1 and NDSTC-1, IL loading even of only 50% of the pore volume leads to a complete loss of the porosity available for nitrogen at 77 K; that is, the partial loading with IL also blocks the entries to the conceptually still open pores. A filling of 100% of the pore volume with ionic liquid leads in all cases to a complete loss of porosity, which indicates a homogeneous filling of pores of the carbons with the (then frozen) ionic liquid and the correctness of the process applied for specific pore volume determination and filling.

A first indication of a special behavior of confined ILs is given by thermal stability. TGA in synthetic air shows that the bulk IL completely decomposes between 200 and 300 °C (Figure 3a). In the pores, the onset point of decomposition remains similar, but the mass loss is much broader and has components up to 600 °C, where also the carbon starts to oxidize (Figure 3b).

A comparison of the carbons with comparable pore structure but different IL content (such as IL-(ND)STC-8-50% and -100%) shows that the mass loss up to 600 °C follows the IL content in the materials. In addition, the residual mass of the loaded microporous carbon material STC-1 at 600 °C is higher as compared to the mesoporous STC-8, indicating that it is mostly the ions in the narrow micropores that condense with the wall material. It should be noticed that minor residual masses (below 10%) are obtained for some of the IL-loaded carbons. This could be related to repolymerization of decomposition products of the ILs within the TGA. The existence of inorganic contaminations can be ruled out by the complete mass loss of the unloaded carbon materials upon heating to 1000 °C under synthetic air.

Another interesting effect is the distinct mass loss at temperatures below 100 °C of the IL-loaded carbon materials. Such mass loss is not observed for the pristine carbons nor for the bulk ionic liquid and of course at a too low temperature to be related to decomposition or water uptake. This stepwise loss indicates that the IL—when confined in the carbon pores—is able to store a significant amount (apparently more than 20% of its own mass) of gases, here the synthetic air atmosphere under which the measurements have been performed. The nanoconfinement of the IL therefore strongly increases its ability to absorb gases, and this is so intense that it is seen already in TGA. Independent of the IL content, nitrogen doping of the carbon increases this effect, and the corresponding mass loss step below 100 °C of the IL-loaded NDSTC materials is larger.

**Figure 5:**
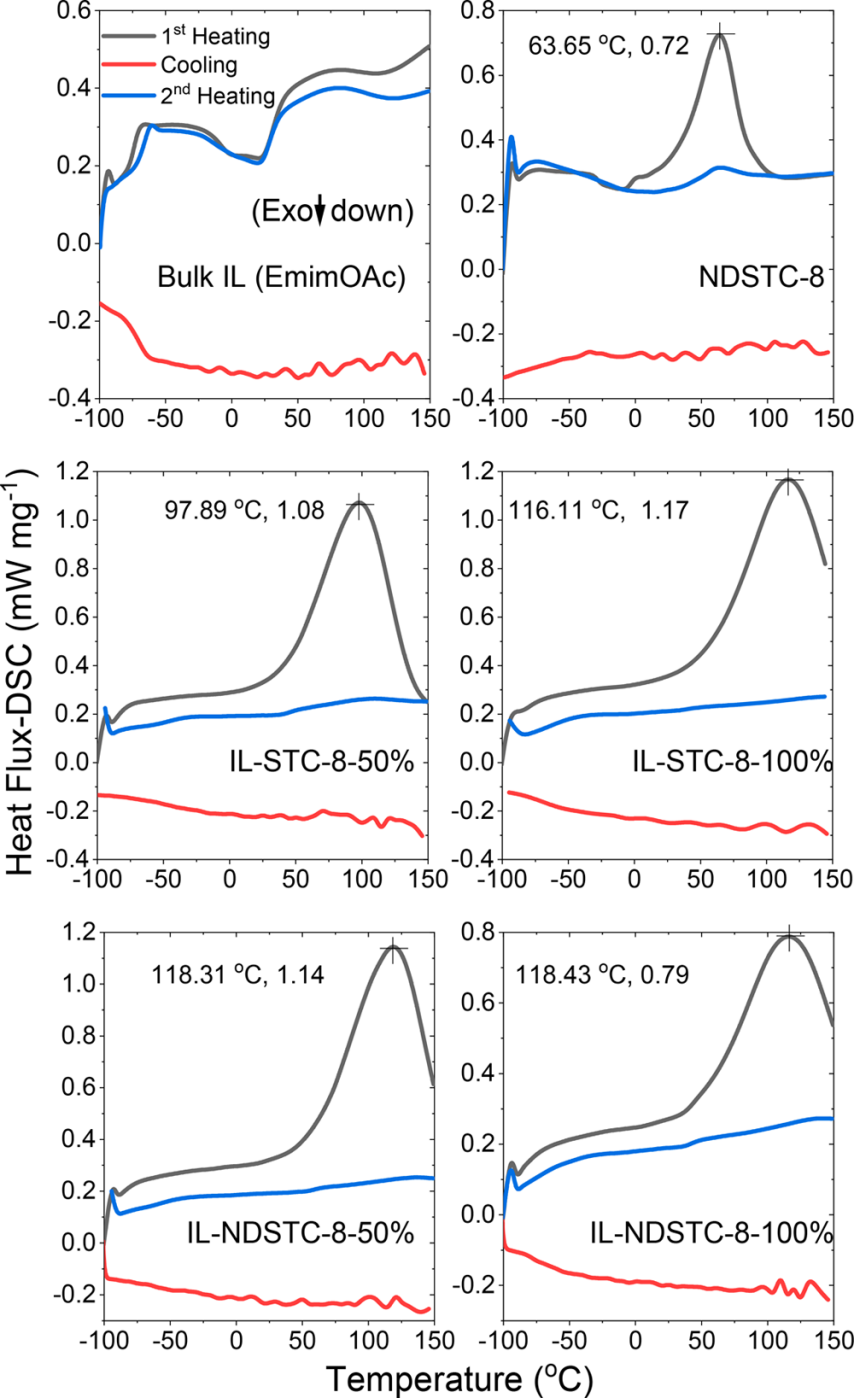
DSC curves of EmimOAc, pristine NDSTC-8, and IL-loaded STC-8 and NDSTC-8 carbons.

Differential scanning calorimetry (DSC) analysis under N_2_ atmosphere has been performed between −100 and +150 °C for the bulk IL, pristine NDSTC-8 carbon, and the IL-loaded STC-8 and NDSTC-8 samples to observe possible phase transitions of the IL (Figure 5). The DSC curve of the nonconfined bulk EmimOAc shows the presence of multiple phase changes and steps, which is typical for ionic liquids during the first heating cycle, and all of these are fully reversible, as can be seen by the similar heat flux profile during the second cycle. No distinct peaks occur during cooling, which might be due to slow reordering kinetics, as has been reported for Emim-based ILs before.^34^ A look at the DSC curves of pore-confined ILs shows neither in the first nor in the second heating significant phase transition signals as observed for the bulk IL. This is attributed to the large number of coordinately unsaturated ions that are in direct contact with the carbon surface. In other words, the IL entities are too small to form a phase with a defined phase transition. This is typical for liquid matter in nanodimensions.

For the pristine NDSTC-8, an endothermic process occurs at ∼64 °C in the first heating cycle, which is likely due to the desorption of residual gases strongly adsorbed in the pores of this heteroatom-doped carbon. In the second cycle this peak almost disappeared, indicating the complete removal of all “desorbable” gas molecules during the first heating cycle. After IL loading, an endothermic peak at higher temperatures is observed. In accordance with the TGA data, this peak likely corresponds to gases present in the pore-confined IL. The shift of this peak to higher temperatures indicates that there is a stronger interaction between gases and IL when confined into carbon pores; that is, the interaction is stronger than in the bulk IL.

At this stage, we can already speculate about the nature of this nonclassical, gigantic extra gas uptake. From supercapacitor experiments it is known that pore confinement of ILs leads to a change of the arrangement of the constituting ions.^32,35^ This is already intuitively clear as the pore size reaches the size of the IL ions, and the incommensurability of pore volume and solvent volume will lead to “frustrated arrangements”, extra “free volume” between solvent molecules and the wall, and essentially also the breakup of the extended, energy-optimized ionic structure otherwise known from the IL bulk liquid. The concept of “free volume” is well elaborated in polymers and the description of the glass transition^36^ and can be also related to the dissolution and permeation of gases through glassy polymer films.^37^ Such free volume sites are too small to allow an IL ion to move in but are big enough to host the comparably small gas molecules. As that, it can be concluded that the mechanism of gas uptake in this confined liquid is similar to the formation of inclusion compounds as they occur during the formation of methane hydrate^4,38^ rather than being a classical dissolution process, which would be only weakly endothermic. This extra dissolution is indeed gigantic, as it concerns an uptake of 20 wt % of inert gases at comparably high operation temperatures. The stepwise weight loss too is typical for the formation of a joint phase with a “melting point”, while adsorption as an activated process can be excluded.

In addition, the very nonpolar dinitrogen molecule provides a comparably low adsorption enthalpy, which makes adsorption rather inefficient at the examined ambient temperatures, and further details of the binding mechanism have to be elaborated. Interestingly, nitrogen uptake in ILs can be also additionally quantified with a classical technique, volumetric nitrogen sorption experiments. This is only possible for liquids with very low vapor pressure, such as ionic liquids. These experiments have been performed at room temperature for the bulk IL and the IL-loaded carbon materials. In all the following cases we will refer to these measurements as “sorption” isotherms, in spite of the fact that the observed uptake is not at surfaces (but in frustrated liquids).

The nitrogen sorption isotherm of the bulk IL (Figure S6) shows that EmimOAc has an uptake of ∼4.2 cm^3^/g at 298 K/273 K at 1 bar nitrogen pressure. This corresponds to a storage capacity of around 5.25 mg N_2_/g_IL_, which is more than 200 times higher than the solubility of nitrogen in water (∼0.02 mg N_2_/g_H2O_). ILs have been described as “porous liquids” before,^39^ and in addition to the partial free volume between the individual ions to be filled, the charged character leads to strong polarization of N_2_ molecules. The nitrogen uptake at 298 K of NDSTC-1 and NDSTC-8 before IL loading is similar and follows the relatively similar micropore volumes (Figure S7a). At a lower adsorption temperature of 273 K, both uptakes slightly increase (Figure S7b), as is typical for the exothermic physisorption. The temperature dependence is more obvious for the purely microporous NDSTC-1.

**Figure 6:**
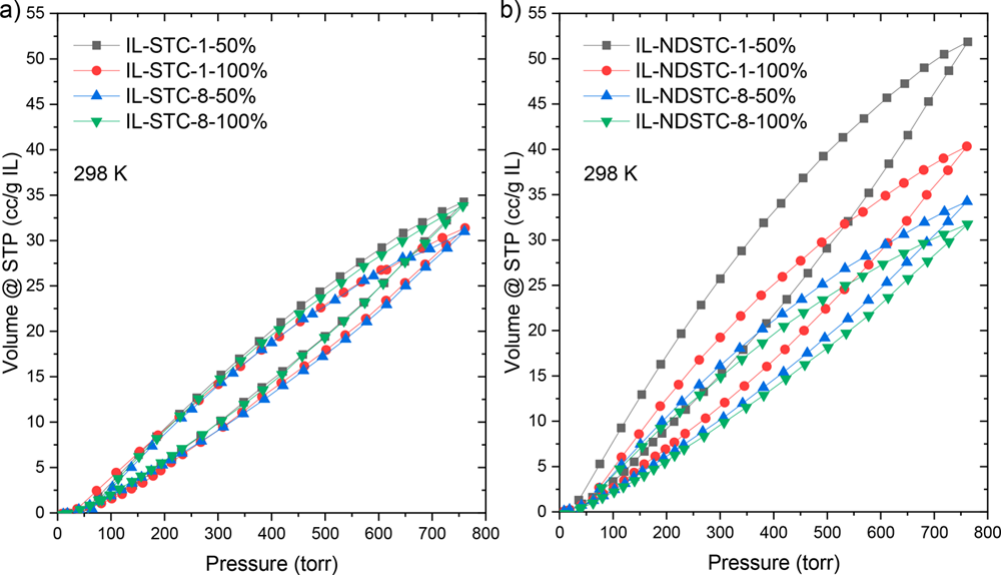
N_2_ sorption isotherms of (a) IL-loaded STC materials and (b) IL-loaded NDSTC materials measured at 298 K. The uptake is normalized to the mass of IL within the samples.

Volumetric nitrogen sorption isotherms and uptakes of the IL-loaded STC carbon materials (Figure 6, Figure S8, and Table 2) show that the nitrogen uptake increases significantly in comparison to the bulk IL when the EmimOAc is confined in the carbon pores. One observes a slight hysteresis over the entire range of pressures, which can be referred to the fact that volume uptake approaches equilibrium slower than surface adsorption from the gas phase, but the uptake remains reversible on the overall time scale of the experiment; that is, the loops are closed. The more than 10-times higher nitrogen uptake of ILs confined into the micropores of IL-NDSTC-1-50% in comparison to the bulk IL is particularly remarkable (Table 2). We remind the reader that this sample had no accessible pore volume detectable by nitrogen physisorption at 77 K, and thus it can be concluded that nitrogen is here absorbed within the IL structure rather than being physisorbed on a nonopen solid surface. The influence of kinetics cannot be strictly ruled out, but on the other hand the shape of the isotherms, including the hysteresis (here taken as a sign of potential kinetic effects), remains comparable to the measurement of the bulk IL.

**Table 2:** N_2_ Uptakes at 1 bar (Normalized to the Content of IL in the Samples) of the IL-Loaded Materials at 273 and 298 K

	N_2_ uptake (cm^3^/g_IL_)	
sample	298 K	273 K
IL-STC-1-50%	34.3	35.6
IL-STC-1-100%	31.4	32.5
IL-STC-8-50%	31.0	27.1
IL-STC-8-100%	33.8	32.4
IL-NDSTC-1-50%	51.9	52.6
IL-NDSTC-1-100%	40.3	40.3
IL-NDSTC-8-50%	34.2	34.7
IL-NDSTC-8-100%	31.8	29.4
EmimOAc	4.3	4.2

Interestingly, if not most importantly, there seems no significant influence of the measurement temperature in the range from 273 to 298 K; that is, this giant nitrogen sorption is due to a nonactivated process. Again it is not adsorption but rather corresponds to the formation of a phase in which nitrogen molecules are embedded in the ionic network and which is stable up to a critical temperature (presumably described by the temperature of the “melting peak” or the gas release found in both DSC and TGA measurements, respectively).

When the experimental N_2_ uptake is normalized to the mass of the carbon materials, it becomes evident that a higher IL loading increases the uptake. This further underlines that N_2_ is absorbed in the volume of the IL rather than adsorbed on the surface of the carbon. In the future, gases with less sorption affinity such as hydrogen or helium or even supercritical nitrogen might be used to further confirm this effect, which should occur independent of the used adsorptive. Furthermore, gases with higher boiling point such as carbon dioxide would also be suitable to investigate the influence of pore confinement of ILs on their gas sorption ability further. However, possible reversibility problems might occur in these cases.

The particular influence of nitrogen doping on the giant gas solubility is more pronounced in the microporous materials STC-1 and NDSTC-1. At both IL-loadings, nitrogen doping leads to a higher N_2_ uptake. As discussed above, there is no distinct change in the pore size and architecture caused by nitrogen doping. It can thus be expected that the changed electron density distribution in the micropore walls changes the adsorption state of the IL ions and thus its structure, which in turn also changes the uptake of nitrogen molecules. Even though a slightly lower pore volume is detected in NDSTC-1 in comparison to STC-1, the uptake of N_2_ relative to the mass of carbon is higher after nitrogen doping. This difference is less pronounced in mesopores, where fewer IL ions are in direct contact with the pore walls. Such a structure change of the IL ions in mesopores can however be promoted by applying external electric fields to the carbons,^29^ and it stays an open question in this paper if application of an electric potential would also modify the nitrogen uptake in the mesopores. For sure, capillary pressure acting on the N_2_ molecules in small pores together with the interactions caused by the generated local electric fields and the nitrogen molecules with significant quadrupole moment do contribute to the remarkable nitrogen uptake even without the application of an electric potential.

## Conclusions and Outlook

A matrix of four carbon materials with meso- and microporosity as well as with and without nitrogen doping has been prepared to investigate the nitrogen uptake within a pore-confined IL, EmimOAc. The nitrogen uptake of the IL-filled carbon pores reaches unexpectedly high values, much higher than even the empty pores, and the mechanism of this gigantic uptake clearly shifts from surface physisorption to absorption. The enhancement of dinitrogen uptake is most pronounced in the micropores of nitrogen-doped carbon materials, where consequently also the structural changes of the IL are maximized. In general, ILs that are located close to carbon pore walls (i.e., that strongly interact) seem to contribute more significantly to partial free volume and thereby loci of dinitrogen absorption.

This study thereby opens up the way to the application of pore-confined ILs as reaction media for the catalytic activation and conversion of small molecules, such as dinitrogen in NRR, at unusual high local concentrations of up to 20 wt %. The further presence of an electric field may induce ordering transitions in the IL ions^32,40^ and thus change the solubility of gas molecules even further. We found the interaction between IL ions and dinitrogen to be strong; that is, it is constant in the examined temperature range, but the gas is rather cooperatively released when reaching a “order–disorder” transition above 60 °C. This dinitrogen–solvent interaction will not only cause the gigantic absorption capacity but might lead to catalytic activation of the otherwise highly stable N_2_ within the dipolar free volume sites between the IL ions and dinitrogen. Likewise, other electrocatalytic conversions could also benefit from this significant change of the physicochemical properties of ILs after pore confinement. Capacitive electrochemical energy storage is another possible field of application into which the changed gas absorption properties of pore-confined ILs can play a role. However, translation of the findings reported in this study to a real application in electrocatalysis or capacitive energy storage needs the development of novel reactor concepts, analytical tools, and a profound understanding of mass transfer in such gas–liquid–solid interfaces. This is part of ongoing investigations. Furthermore, EmimOAc has been chosen in this study for its wide application in combination with nanocarbon materials in electrochemical energy storage applications. ILs with other cation–anion combinations will surely be another possibility to further underline and optimize the findings of our study.

## Supporting Information Available

Experimental details, additional adsorption data as well as electron microscopy images, and elemental analysis data (PDF)

## References

[ref1] Battino R.; Clever H. L. The solubility of gases in liquids. Chem. Rev. 1966, 66, 395–463. 10.1021/cr60242a003.

[ref2] Markham A. E.; Kobe K. A. The Solubility of Gases in Liquids. Chem. Rev. 1941, 28, 519–588. 10.1021/cr60091a003.

[ref3] Wilhelm E.; Battino R.; Wilcock R. J. Low-pressure solubility of gases in liquid water. Chem. Rev. 1977, 77, 219–262. 10.1021/cr60306a003.

[ref4] Borchardt L.; Casco M. E.; Silvestre-Albero J. Methane hydrate in confined spaces: an alternative storage system. ChemPhysChem 2018, 19, 1298–1314. 10.1002/cphc.201701250. 29537620

[ref5] Someya S.; Bando S.; Chen B.; Song Y.; Nishio M. Measurement of CO_2_ solubility in pure water and the pressure effect on it in the presence of clathrate hydrate. Int. J. Heat Mass Transfer 2005, 48, 2503–2507. 10.1016/j.ijheatmasstransfer.2004.12.043.

[ref6] Kilburn D.; Lilly M.; SELF D. A.; Webb F. The effect of dissolved oxygen partial pressure on the growth and carbohydrate metabolism of mouse LS cells. J. Cell Sci. 1969, 4, 25–37. 10.1242/jcs.4.1.25. 5777809

[ref7] Prüße U.; Herrmann M.; Baatz C.; Decker N. Gold-catalyzed selective glucose oxidation at high glucose concentrations and oxygen partial pressures. Appl. Catal., A 2011, 406, 89–93. 10.1016/j.apcata.2011.08.013.

[ref8] Qin Q.; et al. Enhanced electrocatalytic N_2_ reduction via partial anion substitution in titanium oxide-carbon composites. Angew. Chem., Int. Ed. 2019, 58, 13101–13106. 10.1002/anie.201906056. 31257671

[ref9] Foster S. L.; et al. Catalysts for nitrogen reduction to ammonia. Nature Catalysis 2018, 1, 490 10.1038/s41929-018-0092-7.

[ref10] Guo X.; Du H.; Qu F.; Li J. Recent progress in electrocatalytic nitrogen reduction. J. Mater. Chem. A 2019, 7, 3531–3543. 10.1039/C8TA11201K.

[ref11] Qin Q.; Heil T.; Antonietti M.; Oschatz M. Single-Site Gold Catalysts on Hierarchical N-Doped Porous Noble Carbon for Enhanced Electrochemical Reduction of Nitrogen. Small Methods 2018, 2, 6 10.1002/smtd.201800202.

[ref12] Cao N.; Zheng G. Aqueous electrocatalytic N_2_ reduction under ambient conditions. Nano Res. 2018, 11, 2992–3008. 10.1007/s12274-018-1987-y.

[ref13] Sun R.; Hu W.; Duan Z. Prediction of nitrogen solubility in pure water and aqueous NaCl solutions up to high temperature, pressure, and ionic strength. J. Solution Chem. 2001, 30, 561–573. 10.1023/A:1010339019489.

[ref14] Dyson P. J.; Ellis D. J.; Henderson W.; Laurenczy G. A Comparison of Ruthenium-Catalysed Arene Hydrogenation Reactions in Water and 1-Alkyl-3-methylimidazolium Tetrafluoroborate Ionic Liquids. Adv. Synth. Catal. 2003, 345, 216–221. 10.1002/adsc.200390015.

[ref15] Silveira E. T.; et al. The partial hydrogenation of benzene to cyclohexene by nanoscale ruthenium catalysts in imidazolium ionic liquids. Chem. - Eur. J. 2004, 10, 3734–3740. 10.1002/chem.200305765. 15281157

[ref16] Suarez P. A.; Dullius J. E.; Einloft S.; De Souza R. F.; Dupont J. The use of new ionic liquids in two-phase catalytic hydrogenation reaction by rhodium complexes. Polyhedron 1996, 15, 1217–1219. 10.1016/0277-5387(95)00365-7.

[ref17] Lai F.; et al. Breaking the Limits of Ionic Liquid-Based Supercapacitors: Mesoporous Carbon Electrodes Functionalized with Manganese Oxide Nanosplotches for Dense, Stable, and Wide-Temperature Energy Storage. Adv. Funct. Mater. 2018, 28, 1801298 10.1002/adfm.201801298.

[ref18] Silvester D. S.; Compton R. G. Electrochemistry in room temperature ionic liquids: a review and some possible applications. Z. Phys. Chem. 2006, 220, 1247–1274. 10.1524/zpch.2006.220.10.1247.

[ref19] Yan R.; et al. Ordered Mesoporous Carbons with High Micropore Content and Tunable Structure Prepared by Combined Hard and Salt Templating as Electrode Materials in Electric Double-Layer Capacitors. Advanced Sustainable Systems 2018, 2, 1700128 10.1002/adsu.201700128.

[ref20] Anthony J. L.; Anderson J. L.; Maginn E. J.; Brennecke J. F. Anion effects on gas solubility in ionic liquids. J. Phys. Chem. B 2005, 109, 6366–6374. 10.1021/jp046404l. 16851709

[ref21] Ejigu A.; Walsh D. A. The role of adsorbed ions during electrocatalysis in ionic liquids. J. Phys. Chem. C 2014, 118, 7414–7422. 10.1021/jp411730z.

[ref22] Finotello A.; Bara J. E.; Camper D.; Noble R. D. Room-temperature ionic liquids: temperature dependence of gas solubility selectivity. Ind. Eng. Chem. Res. 2008, 47, 3453–3459. 10.1021/ie0704142.

[ref23] Holbrey J.; Seddon K. Ionic liquids. Clean Technol. Environ. Policy 1999, 1, 223–236. 10.1007/s100980050036.

[ref24] Marciniak A. The solubility parameters of ionic liquids. Int. J. Mol. Sci. 2010, 11, 1973–1990. 10.3390/ijms11051973. 20559495 PMC2885087

[ref25] Zhang G.-R.; Straub S.-D.; Shen L.-L.; Hermans Y.; Schmatz P.; Reichert A. M.; Hofmann J. P.; Katsounaros I.; Etzold B. J. M. Probing CO_2_ Reduction Pathways in Copper Catalysts using Ionic Liquid as a Chemical Trapping Agent. Angew. Chem., Int. Ed. 2020, 59, 18095–18102. 10.1002/anie.202009498. PMC7589334 32697377

[ref26] Wilkes J. S. A short history of ionic liquids—from molten salts to neoteric solvents. Green Chem. 2002, 4, 73–80. 10.1039/b110838g.

[ref27] Zhou F.; et al. Electro-synthesis of ammonia from nitrogen at ambient temperature and pressure in ionic liquids. Energy Environ. Sci. 2017, 10, 2516–2520. 10.1039/C7EE02716H.

[ref28] Kang C. S. M.; Zhang X.; MacFarlane D. R. Synthesis and Physicochemical Properties of Fluorinated IonicLiquids with High Nitrogen Gas Solubility. J. Phys. Chem. C 2018, 122, 24550–24558. 10.1021/acs.jpcc.8b07752.

[ref29] Schutjajew K.; Yan R.; Antonietti M.; Roth C.; Oschatz M. Effects of Carbon Pore Size on the Contribution of Ionic Liquid Electrolyte Phase Transitions to Energy Storage in Supercapacitors. Front. Mater. 2019, 6, 65 10.3389/fmats.2019.00065.

[ref30] Eggenhuisen T. M.; van Steenbergen M. J.; Talsma H.; de Jongh P. E.; de Jong K. P. Impregnation of mesoporous silica for catalyst preparation studied with differential scanning calorimetry. J. Phys. Chem. C 2009, 113, 16785–16791. 10.1021/jp905410d.

[ref31] Morishige K.; Kawano K. Freezing and melting of water in a single cylindrical pore: The pore-size dependence of freezing and melting behavior. J. Chem. Phys. 1999, 110, 4867–4872. 10.1063/1.478372.

[ref32] Yan R.; Antonietti M.; Oschatz M. Toward the experimental understanding of the energy storage mechanism and ion dynamics in ionic liquid based supercapacitors. Adv. Energy Mater. 2018, 8, 1800026 10.1002/aenm.201800026.

[ref33] Walczak R.; Kurpil B.; Savateev A.; Heil T.; Schmidt J.; Qin Q.; Antonietti M.; Oschatz M. Template- and Metal-Free Synthesis of Nitrogen-Rich Nanoporous “Noble” Carbon Materials by Direct Pyrolysis of a Preorganized Hexaazatriphenylene Precursor. Angew. Chem., Int. Ed. 2018, 57, 10765–10770. 10.1002/anie.201804359. 29882376

[ref34] Weingarth D.; Drumm R.; Foelske-Schmitz A.; Kötz R.; Presser V. An electrochemical in situ study of freezing and thawing of ionic liquids in carbon nanopores. Phys. Chem. Chem. Phys. 2014, 16, 21219–21224. 10.1039/C4CP02727B. 25201074

[ref35] Futamura R.; Iiyama T.; Takasaki Y.; Gogotsi Y.; Biggs M. J.; Salanne M.; Ségalini J.; Simon P.; Kaneko K. Partial breaking of the Coulombic ordering of ionic liquids confined in carbon nanopores. Nat. Mater. 2017, 16, 1225–1232. 10.1038/nmat4974. 28920938 PMC5702543

[ref36] Napolitano S.; Glynos E.; Tito N. B. Glass transition of polymers in bulk, confined geometries, and near interfaces. Rep. Prog. Phys. 2017, 80, 036602 10.1088/1361-6633/aa5284. 28134134

[ref37] Corrado T.; Guo R. Macromolecular design strategies toward tailoring free volume in glassy polymers for high performance gas separation membranes. Molecular Systems Design & Engineering 2020, 5, 22–48. 10.1039/C9ME00099B.

[ref38] Borchardt L.; et al. Illuminating solid gas storage in confined spaces–methane hydrate formation in porous model carbons. Phys. Chem. Chem. Phys. 2016, 18, 20607–20614. 10.1039/C6CP03993F. 27412621

[ref39] Zhang S.; Dokko K.; Watanabe M. Porous ionic liquids: synthesis and application. Chemical Science 2015, 6, 3684–3691. 10.1039/C5SC01374G. 28706714 PMC5496189

[ref40] Antonietti M.; Chen X.; Yan R.; Oschatz M. Storing electricity as chemical energy: beyond traditional electrochemistry and double-layer compression. Energy Environ. Sci. 2018, 11, 3069–3074. 10.1039/C8EE01723A.

