# Supplementary material for: “Giant” Nitrogen Uptake in Ionic Liquids Confined in Carbon Pores
Source: J Am Chem Soc. 2021 Jun 15;143(25):9377–84. doi: 10.1021/jacs.1c00783 (PMC8251693; doi:10.1021/jacs.1c00783)
Supplement: Supplementary file 1 — ja1c00783_si_001.pdf [file ja1c00783_si_001.pdf]

Supporting Information

"Giant" nitrogen uptake in ionic liquids confined in carbon pores

Ipek Harmanli,[a,b] Nadezda V. Tarakina,[a] Markus Antonietti,[a] Martin Oschatz[a,b,+,*]

[a] Max Planck Institute of Colloids and Interfaces, Department of Colloid Chemistry, Research Campus Golm, Am Mühlenberg 1, 14476 Potsdam, Germany.

*Corresponding author email: martin.oschatz@mpikg.mpg.de

[b] University of Potsdam, Institute of Chemistry, Karl-Liebknecht-Str. 24-25, D-14476 Potsdam, Germany.

[+] current address: Friedrich-Schiller-University Jena, Institute for Technical Chemistry and Environmental Chemistry, Center for Energy and Environmental Chemistry Jena (CEEC Jena), Philosophenweg 7a, Jena 07743, Germany.

Experimental Section

Synthesis of STC and NDSTC materials and loading of EmimOAc. 5 g Sucrose and 5 g $ZnCl_2$ salt template were dissolved in 30 mL water. Then, 0.53 g concentrated sulfuric acid was added to the solution and the mixture was then treated for 6 h at 100°C and for another 6h at 160°C in a petri dish under air atmosphere for polycondensation of the sucrose. The polymerized carbohydrate was carbonized at 900°C for 2 h under N_2 flow in a horizontal furnace (heating rate 150°C h^{-1}). In order to remove the remaining salt template, the carbonized product was stirred in 1 L of 1 M aqueous HCl solution for 3 days. The solution has been exchanged every day. In the final step, the carbons were washed several times with water by vacuum filtration. The dried carbon powder is labeled as STC-1. Following the same synthesis route, STC-8 was also synthesized by changing just the amount of used salt template (the mass of $ZnCl_2$ was 40g for STC-8).

For nitrogen-doping of STCs, cyanamide (CH_2N_2) was impregnated into the carbons with a carbon:cyanamide weight ratio of 1:2. An aqueous cyanamide solution was prepared with respect to total pore volumes (TPV) of the STCs. This aqueous solution was added to the carbon powders by incipient wetness impregnation in mortar. The loaded powder was dried in an oven at 60°C overnight. Cross-condensation of the cyanamide was carried out at 900°C for 2 h under N_2 flow in a horizontal furnace (heating rate 120°C h^{-1}).
For the loading of EmimOAC (1-Ethyl-3-methylimidazolium acetate, 95%, purchased from from Iolitec GmbH) into the carbon pores, the volume of IL corresponding to 50 or 100 vol.% of the carbon pore volume was dissolved in 1 mL acetonitrile. After addition of the carbon material, the solution was stirred overnight at room temperature until the acetonitrile was completely evaporated. Finally, 8 IL-doped carbon samples (denoted as IL-STC-1-50%, IL-STC-1-100%, IL-STC-8-50%, IL-STC-8-100%, IL-NDSTC-1-50%, IL-NDSTC-1-100%, IL-NDSTC-1-50%,IL-NDSTC-1-100% according to the IL-loading ratio) were obtained and further dried into a vacuum oven at 60°C.

Structural Characterization and Adsorption Measurements. N_2 sorption measurements were carried out with a Quadrasorb apparatus (Quantachrome Instruments) at 77 K (under liquid nitrogen), 298 K (ambient temperature) and 273 K (ice water bath). Before the all measurements, 40-60 mg of sample were outgassed for 20 h under vacuum at 150°C (non IL-loaded samples) and room temperature (IL-loaded samples). In order to calculate the specific surface area of the samples (SSA_{BET}) from the physisorption isotherms at 77 K, the multipoint BET (Brunauer–Emmett–Teller) model was applied ($p/p_0 = 0.05$-0.2). The total pore volumes were determined at $p/p_0 = 0.95$. Moreover, by using the QSDFT method (quenched solid density functional theory, adsorption branch kernel), pore size distributions (PSD) were determined for N_2 adsorbed on carbons. Gas uptakes of IL-loaded samples are normalized to the nominal content of ionic liquid or carbon after mixing.
Transmission electron microscopy studies were carried out using a TEM-EM-912 Zeiss Omega with a LaB_6 cathode operated at 120 kV. Scanning transmission electron microscopy and electron energy spectroscopy data were collected on a double Cs corrected JEOL JEM-ARM200F (S)TEM operated at 80 kV and equipped with a cold-field emission gun and a Gatan Quantum GIF spectroscopy system.
The scanning electron microscopy was carried out with a Zeiss Gemini LEO 1550 microscope equipped with a FEG gun. The images were collected at 3kV using In-Lens and ETD secondary electron detectors. EDX data were collected at 10 kV using an Oxford Instruments MAX 80mm^2 SDD detector.

The carbon and nitrogen content of the samples was investigated by elemental analysis with a vario MICRO cube CHNOS Elemental Analyzer (Elementar Analysensysteme GmbH, Langenselbold). Oxygen contents were not specifically analyzed. Besides carbon and nitrogen, traces of sulfur were also detected in the STC and NDSTC materials (between 2 and 6.3 wt.%). TGA measurements have been performed using a thermo microbalance (TG-209-F1-Libra, Netzsch, Selb, Germany). A platinum crucible was used for the measurement of 10 (±1) mg of samples in a nitrogen flow of 20 mL/ min and a purge flow of 20 mL/ min (oxygen flow 10 mL/ min) as under syn-air at a heating rate of 30°C/ 10 (K/min)/ 1000°C.
DSC measurements have been performed with a Differential Scanning Calorimeter, DSC 204 F1 Phoenix (Netzsch, Selb, Germany). An aluminum pan with a pierced lid was used for the measurement of 10 (±1) mg of the sample with a heating/cooling rate of 10 K/min in range between - 100°C / 10 (K/min)/ 150°C at 5000µV. Then, data for both measurement techniques have been recorded and analyzed by the Proteus (software 6.1.0 and 8.0.0).

Supplementary Figures

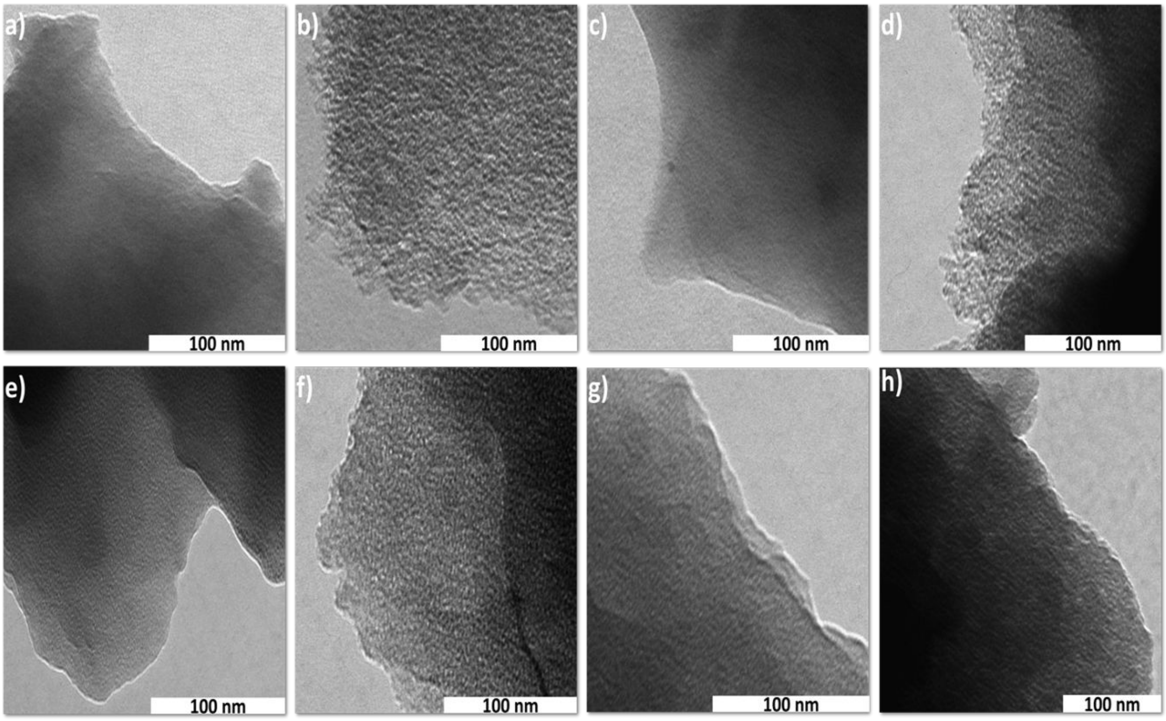

Figure S1. TEM images of a) STC-1, b) STC-8, c) NDSTC-1, d) NDSTC-8, e) IL-STC-1-50%, f) IL-STC-1-100%, g) IL-NDSTC-8-50%, h) IL-NDSTC-8-100%.

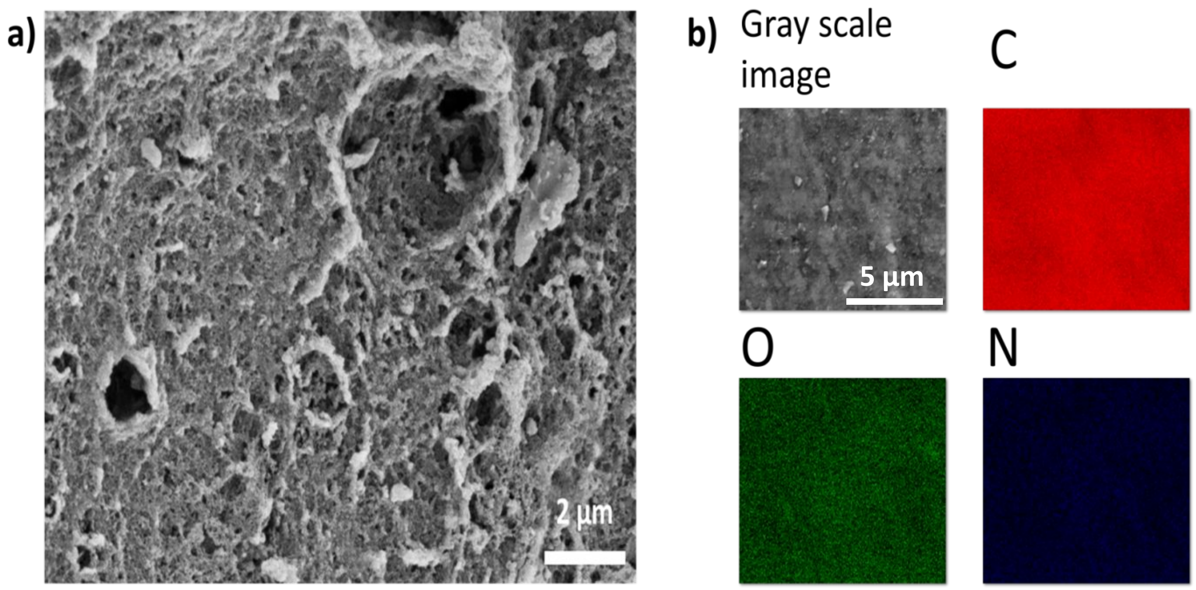

Figure S2. a) SEM image and b) EDX mapping analysis of NDSTC-8.

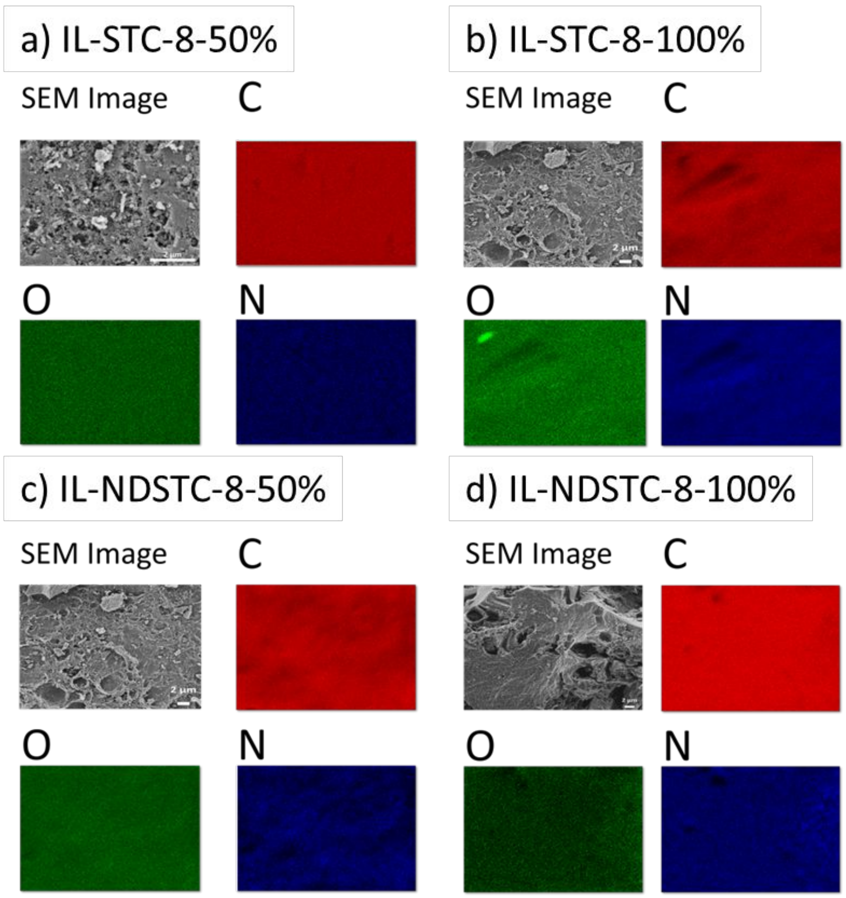

Figure S3. SEM images and EDX mapping analysis of a) IL-STC-8-50%, b) IL-STC-8-100%, c) IL-NDSTC-8-50%, and d) IL-NDSTC-8-100%.

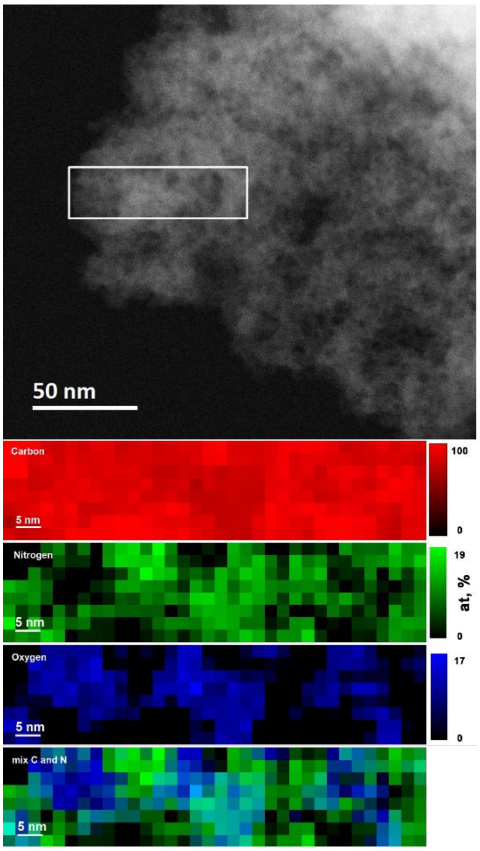

Figure S4. High-resolution annular dark- field STEM image of IL-NDSTC-8-100%. The white rectangle marks the area from which corresponding EELS maps were taken.

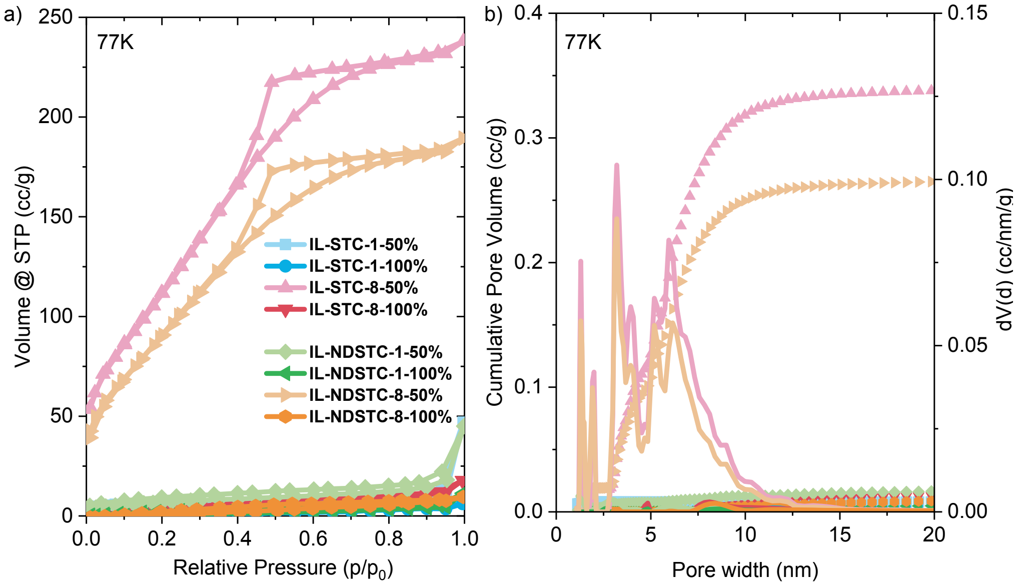

Figure S5. a) N_2 physisorption isotherms measured at 77 K and b) corresponding cumulative (line with symbols) and differential (lines without symbols) QSDFT pore size distributions of the IL-loaded carbon materials.

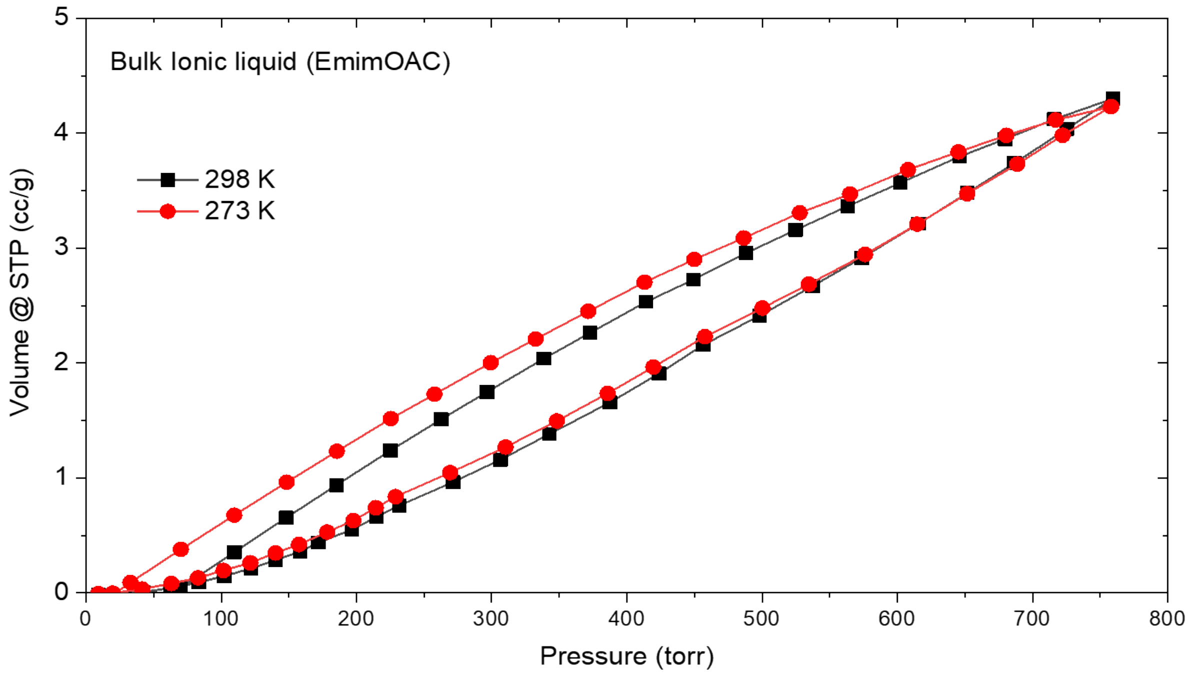

Figure S6. N_2 sorption isotherms of the bulk EmimOAc measured at 298 K and 273 K.

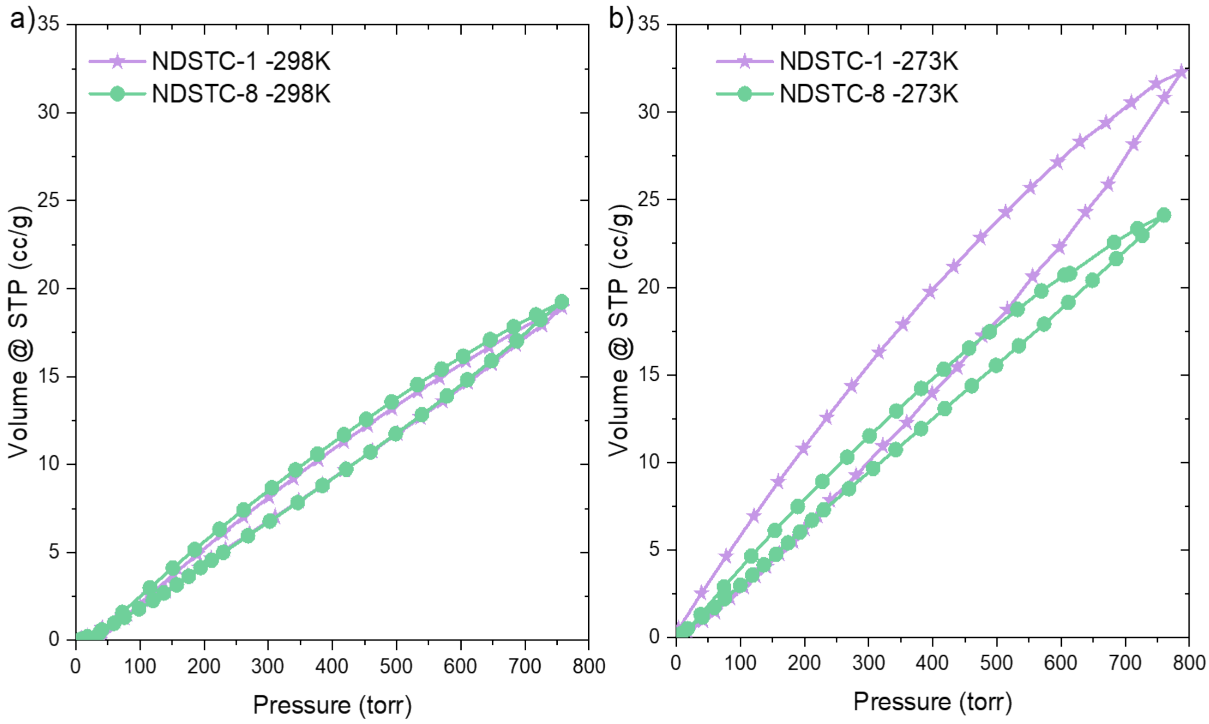

Figure S7. N_2 physisorption isotherms of NDSTC-1 and NDSTC-8 measured at a) 298 K and b) 273 K.

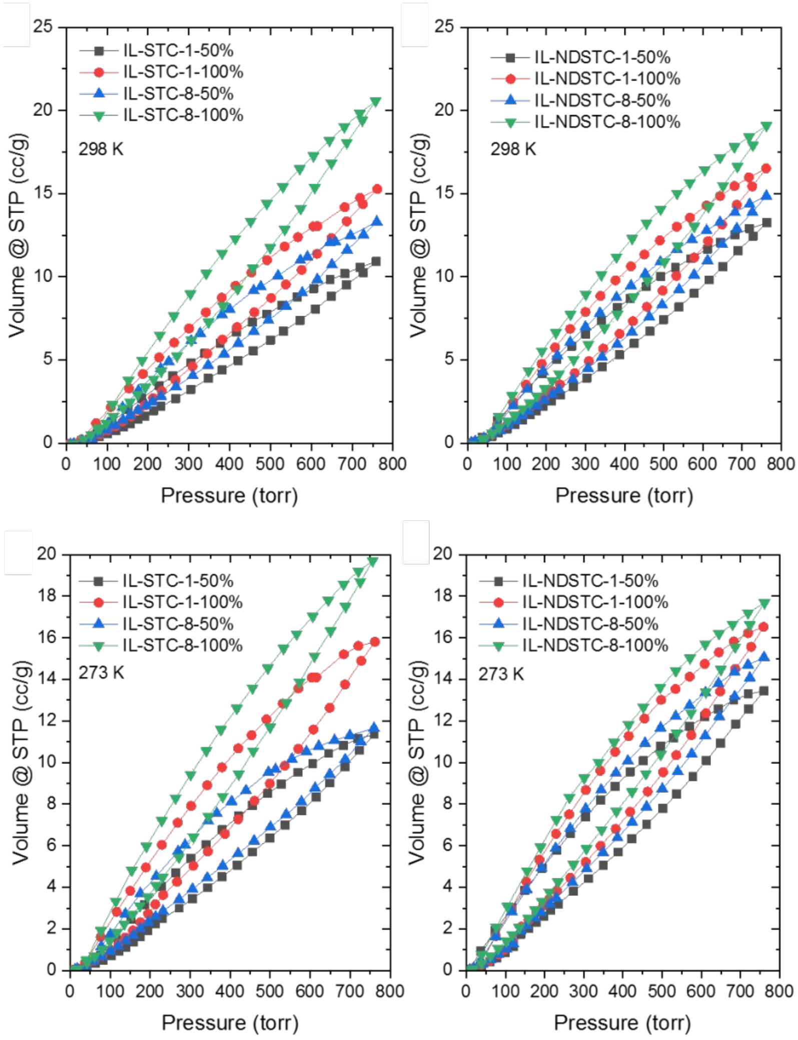

Figure S8. N_2 sorption isotherms of IL-loaded STC materials (left) and IL-loaded NDSTC materials (right) measured at 298 K (top) and 273 K (bottom).

Supplementary Tables

Table S1. Elemental analysis and EDX data summary of selected IL-loaded STC and NDSTC materials.

Sample	Elemental Analysis			EDX	
	N (wt.-%)	C (wt.-%)	C/N	N (wt.-%)	C (wt.-%)
IL-STC-1-50%	4.8	81.2	17.0	-	-
IL-STC-1-100%	6.9	73.4	10.6	-	-
IL-STC-8-50%	6.3	67.6	10.7	9.7	86.1
IL-STC-8-100%	9.1	59.0	6.5	10.7	84.2
IL-NDSTC-8-50%	8.2	66.7	8.1	7.6	89.2
IL-NDSTC-8-100%	9.8	61.2	6.2	13.2	83.4

Table S2. N_2 uptakes at 1 bar (normalized to the content of carbon in the samples) of the IL-loaded materials at 273 K and 298 K.

Sample	N_2 uptake (cm^3/g_{Carbon})	
	298 K	273 K
IL-STC-1-50%	16.0	16.7
IL-STC-1-100%	29.8	30.8
IL-STC-8-50%	23.3	20.4
IL-STC-8-100%	52.4	50.2
IL-NDSTC-1-50%	17.8	18.1
IL-NDSTC-1-100%	28.0	28.0
IL-NDSTC-8-50%	26.2	26.6
IL-NDSTC-8-100%	47.8	44.3
NDSTC-1	18.9	32.3
NDSTC-8	19.3	24.1
